# Comparison of hip fracture incidence and trends between Germany and Austria 1995-2004: An epidemiological study

**DOI:** 10.1186/1471-2458-10-46

**Published:** 2010-01-29

**Authors:** Eva Mann, Gabriele Meyer, Burkhart Haastert, Andrea Icks

**Affiliations:** 1 General Practice and Institute for Health Services Research, Rankweil, Austria; 2 Faculty of Medicine, Institute of Nursing Science, Witten/Herdecke University, Witten, Germany; 3 mediStatistica, Neuenrade, Germany; 4 Faculty of Public Health, Department of Epidemiology and International Public Health, School of Public Health, Bielefeld University, Bielefeld, Germany; and North-Rhine Westfalian Chamber of Physicians, Düsseldorf, Germany

## Abstract

**Background:**

Several studies evaluated variations in hip fracture incidences, as well as trends of the hip fracture incidences. Comparisons of trends are lacking so far. We compared the incidence rates and, in particular, its trends between Austria and Germany 1995 to 2004 analysing national hospital discharge diagnosis register data.

**Methods:**

Annual frequencies of hip fractures and corresponding incidences per 100,000 person years were estimated, overall and stratified for sex and age, assuming Poisson distribution. Multiple Poisson regression models including country and calendar year, age and sex were used to analyse differences in incidence and trend. The difference of annual changes between the two countries was explored using an interaction term (calender year * country).

**Results:**

Overall, the increase of hip fracture risk was 1.31 fold higher (95% CI 1.29-1.34) in Austria compared to Germany, adjusted for age, sex, and calendar year. The risk increase was comparable for both sexes (males: RR 1.35 (1.32-1.37), females: RR 1.31 (1.29-1.33)). Hip fracture trend from 1995 to 2004 indicates an increase in both countries without a statistically significant difference between Austria and Germany (interaction term: p = 0.67).

**Conclusion:**

In this study comparing hip fracture incidences and its trend using pooled data, the incidence in Austria was 30% higher compared to its neighbouring country Germany. For both countries a similar increasing trend of hip fracture incidence over the 10-year study period was calculated. The results need confirmation by other studies.

## Background

Hip fractures are a major public health concern due to their negative impact on health outcomes, quality of life, and costs.

Worldwide hip fracture projection has suggested approximately 1.6 million hip fractures per year. The number is assumed to increase to 4.5 million [1] or even to 6.3 million [2] in 2050.

International analyses indicate large variation in the hip fracture incidence rate throughout countries [3]. Bacon et al. (1996) [4] suggest a tenfold variation with the largest numbers in Scandinavian countries [5,6]. Recent studies have also reported considerable variations throughout geographical regions in several countries [7-9]. Conflicting results on time trends in hip fracture incidence have also been published indicating an increase [10-13], a levelling off, or even a decrease [14-18]. Some studies found a sex-specific difference in hip fracture incidence trends [19-21].

Studies comparing the hip fracture incidence using the same methods and pooled original data are rare. To the best of our knowledge, there are no direct country comparisons of the hip fracture trend.

We recently analysed the country-specific trends of hip fractures in Austria and Germany [22,23]. The aim of the study reported here was to compare the hip fracture incidence rates from 1995 to 2004 between Austria and Germany and to compare the annual trends over the whole study period.

## Methods

### Populations and variables

We used data from the national hospital discharge diagnosis registers provided by the Austria Statistics [24] and the German Federal Statistical Office [25] (data available on personal request). Each hospital admission is registered alongside the patient's age, sex, patient's residence, length of stay, and diagnosis (ICD).

In Austria, the register covers data on hospital discharges from all hospitals since 1989. In Germany, the register covers data since 1992 from more than 99% of all German hospitals. Diagnoses are coded using the International Classification of Diseases ICD 9 (hip fracture: diagnosis 820) up to 1999 in Germany and up to 2000 in Austria [26] and ICD 10 (diagnosis S72.0, S72.1 and S72.2) since 1999 in Germany and 2000 in Austria [27], respectively. The period 1995 to 2004 was chosen since it covers nationwide data of both countries [22,23].

Population characteristics are provided by Austria Statistics for the Austrian population and the National Office of Statistics for the German Population.

### Statistical analysis

Overall and separately for Austria and Germany, we estimated annual frequencies of hip fractures and corresponding incidences per 100,000 person years (PYs) and 95% confidence intervals (95% CIs), overall and stratified for sex and age (0-59 years: 10 year strata; 60-84 years: 5 year strata; ≥ 85 years: last stratum), assuming Poisson distribution. Overall incidence rates were standardised with respect to age and sex according to the European population in 2006 (27 countries, EUROSTAT) as standard population [28]. Corresponding standardised risk ratios (SRRs) and 95% CIs comparing Austria and Germany were estimated.

To analyse the hip fracture incidence and the incidence trend from 1995 to 2004 in Austria and Germany and its difference, we used the pooled data from both countries and multiple Poisson regression models including the incidence of hip fractures (log-persons years as offset in the model specification) as the dependent variable and country (Austria versus Germany), calendar year (ordinal), age (same classes as above) and sex as independent variables. The results of the regression model are adjusted for age and sex. Two-sided 95% CIs of incidence rate ratios (IRRs) were estimated based on the profile likelihood function. An interaction term (calender year * country) was included into the Poisson model to investigate the difference of annual changes between the two countries. In addition, Poisson models were stratified by sex. To take into account overdispersion, all Poisson regressions were performed with DSCALE adjustment. The Poisson models were fitted based on count data stratified by state, year, and sex-age class. The level of significance was 5%. All statistical tests were 2-sided. The Statistical Analysis System SAS (SAS for XP PRO, Release 9.2 TS1 M0, SAS Institute Inc. Cary, NC, USA) was used for statistical analyses.

## Results

### Populations

The total number of the Austrian population increased from 7.943 million in 1995 to 8.140 million in 2004. In Austria 23.6% in 1995 and 22.3% in 2004 were 19 years and older, 56.7% in 1995 and 56.0% in 2004 were in the age of 20 to 59 years, 19.7% in 1995 and 22.3% in 2004 were in the age of 60 years and older [29].

The total number of the German population increased from 81.817 million in 1995 to 82.501 million in 2004. In 1995, 21.5% were 19 years and older, 57.4% were 20 to 59 years, and 21.0% 60 years and older compared to figures in 2004 of 20.3%, 54.8% and 24.9%, respectively [30].

### Incidence of hip fractures

When standardised to the 2006 European population 2006, the overall hip fracture incidence rate (IR) in Austria was 184.4 (95% CI 181.2-187.6) per 100,000 PYs in 1995 and 197.2 (95% CI 194.1-200.4) in 2004. In comparison, the overall standardised IR in Germany was 140.7 (95% CI 139.8-141.5) per 100,000 PYs in 1995 and 150.4 (149.6-151.2) per 100,000 PYs in 2005.

**Figure 1:**
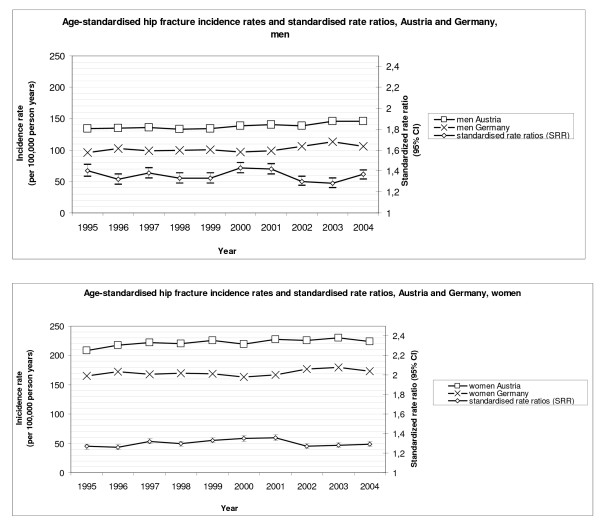
**Incidence rates (IRs) and standardised rate ratios (SRRs) of hip fractures 1995-2004: Austria versus Germany, men and women**.

Figure 1 displays the standardised hip fracture IRs and also the standardised risk ratios (SRRs) for Austrian and German males and females from 1995 to 2004.

Using the pooled data and regression analysis, the increase of hip fracture risk was 1.31 fold increased in Austria compared to Germany (95% CI 1.29-1.34, adjusted for age, sex, and calender year). The risk increase was comparable in both sexes (males: 1.35 (95% CI 1.32-1.37), females: 1.31 (95% CI 1.29-1.33)). The higher hip fracture risk in Austria was statistically significant in all age-sex-strata, except the age group up to 9 years in males, and from 10 to 39 years in females (Poisson regression, adjusted for calendar year) (table 1).

**Table 1:** Incidence rate ratios (IRRs) of hip fractures: Austria versus Germany and time trend (1995-2004)

	Men		Women	
**Age, years**	**Austria vs Germany, IRR (95% CI)**	**Annual change, IRR (95% CI)**	**Austria vs Germany, IRR (95% CI)**	**Annual change, IRR (95% CI)**

0-9	1.11 (0.87-1.40)	0.92 (0.90-0.94)**	1.29 (1.01-1.63)*	0.90 (0.88-0.93)**

10-19	1.41 (1.23-1.61)**	0.95 (0.94-0.97)**	1.18 (0.94-1.45)	0.93 (0.90-0.95)**

20-29	1.17 (1.02-1.32)*	0.95 (0.94-0.97)**	1.11 (0.87-1.39)	0.95 (0.93-0.98)**

30-39	1.17 (1.06-1.29)**	0.98 (0.97-0.99)**	0.98 (0.86-1.13)	0.97 (0.96-0.98)**

40-49	1.22 (1.12-1.32)**	1.00 (1.00-1.01)	1.24 (1.13-1.36)**	0.99 (0.98-1.00)*

50-59	1.34 (1.26-1.42)**	1.00 (0.99-1.00)	1.27 (1.19-1.36)**	1.00 (0.99-1.00)

60-64	1.43 (1.34-1.53)**	1.02 (1.01-1.02)**	1.36 (1.27-1.46)**	0.99 (0.98-1.00)*

65-69	1.39 (1.30-1.48)**	1.00 (0.99-1.01)	1.23 (1.17-1.30)**	0.97 (0.97-0.98)**

70-74	1.38 (1.31-1.46)**	1.01 (1.00-1.02)**	1.19 (1.14-1.24)**	0.99 (0.99-0.99)**

75-79	1.42 (1.34-1.50)**	1.01 (1.01-1.02)**	1.29 (1.23-1.35)**	1.02 (1.01-1.02)**

80-84	1.26 (1.19-1.35)**	1.00 (1.00-1.01)	1.29 (1.22-1.36)**	1.00 (1.00-1.01)

≥ 85	1.40 (1.33-1.46)**	1.03 (1.03-1.04)**	1.36 (1.31-1.43)**	1.02 (1.02-1.03)**

Pooled data from Austria and Germany; Poisson regression (calendar year: ordinal) stratified by sex and age classes.

* p < 0.05, ** p < 0.01

Overall, we found an annual increase of 1% (IRR 1.01 (95% CI 1.01-1.01); adjusted for age, sex and country, without differences between males and females (males: IRR 1.01 (95% CI 1.01-1.01), IRR females: 1.01 (1.01-1.01), adjusted for age and country). For the whole study period, the incidence increased by about 7% (IRR 1995-2004: 1.07; 95% CI 1.05-1.09). The stratum-specific annual changes are displayed in table 1. There was a decrease in younger age groups, and an increase in higher age groups (Poisson regression, adjusted for country).

We did not find a statistically significant difference in the hip fracture trends between Austria and Germany (interaction term: p = 0.67). Sex-specific interactions between calendar year and country were also not statistically significant (males: p = 0.07, females: p = 0.96). Since this might suggest an interaction between calendar year and country in males we additionally present the sex-country stratified results of the corresponding Poisson models (adjusted for age): Austria, males, IRR 1995-2004 (95% CI): 1.02 (0.97-1.07); Germany, males, IRR 1995-2004 (95% CI): 1.09 (1.06-1.11); Austria, females, IRR 1995-2004 (95% CI): 1.07 (1.03-1.10); Germany, females, IRR 1995-2004 (95% CI): 1.07 (1.05-1.09).

## Discussion

We compared hip fracture trends between Austria and Germany using the same methods and pooling original data.

Remarkably, during the 10-year study period the standardised overall incidence rates of hip fracture are about 30% higher in Austria compared to Germany. Despite this constantly higher hip fracture risk in Austria, the incidence similarly increased in both countries between 1995 and 2004. There might be a slight difference between the countries' trends in males. However, interaction was not statistically significant.

The reason for the pronounced difference in hip fracture incidence between the two neighbouring countries alongside a similar incidence trend is unknown so far. Several influencing conditions could be hypothesised. However, interpretation is difficult since valid data covering the whole study period are lacking. Different national drug policies affecting prescription of drugs inducing osteoporosis as well as treating osteoporosis may contribute to the difference between the two countries.

Actually, prescription prevalence of corticoids is higher in Austria compared to Germany, but this is also the case for bisphosphonates (IMS Health [31], personal communication). Concerning raloxifen and hormone replacement therapy the lack of a national drug registration in Austria prohibits comparison. However, individual-linked data for a longer time period are required to draw any conclusion on the possible effect of these drugs. Two recently published studies about hip fracture long-term trends in the US and Canada point out that the impact of antiosteoporotic drug treatment on trends might be overestimated as the decline of hip fracture incidence rates since 1985 in Canada prevailed the market release of bisphosphonates and the decreasing trend after 1995 in the US is only partially attributable to antiosteoporotic drugs [32,33]. Both authors speculate other causes like life style factors including increased body weight, better awareness of falls and osteoporosis or a birth cohort effect to exceed the influence of drugs. All these factors may play a role to explain our findings. According to an analysis of the European Association for the Study of Obesity, prevalence of obesity in German males and females is suggested to be higher compared to Austria [34]. However, we could not identify any other relevant population characteristics' factor expected to explain the difference. In both countries the majority of the population is of Caucasian origin and the proportion of migrants is only insignificantly higher in Austria [35]. The socioeconomic situation is comparable as indicated by the gross domestic product per person (GDP) (2005: GDP per head $37,330 in Austria compared to $33,800 in Germany). The population of both countries equally suffered from starvation and bad nutritional conditions during the World Wars. In addition, it can be assumed that other lifestyle factors including smoking and sedentary behaviour as well as car accidents and risk behaviour might be similar in the two countries, although data are lacking.

The proportion of nursing home residents is also comparable between Austria and Germany (less than 1%). This population has a pronounced hip fracture risk [36]. In both countries no nationwide fall prevention programme has been implemented. Unfortunately, valid data on accidents in the elderly are lacking. Winter conditions in Austria may contribute to the higher hip fracture incidence. The increasing incidence trends in both countries are in contrast to other countries, where a levelling-off or even a decline has been observed [14-18,32,33].

Future studies are needed comparing representative cohorts in Austria and Germany and analysing the underlying causes of hip fractures and socio-demographic determinants including circumstances of falls, applied accidental fall and fracture prevention strategies, and osteoporosis prevention and treatment regimes.

Our study has strengths. A direct comparison of Austrian and German data was feasible since statistical methods of the national register-based data were the same for both countries and pooled original data were analysed. The hospital diagnosis register of both countries covers all hospital admissions due to hip fracture.

Limitations of our study have to be considered. The hospital discharge register provides cases, rather than patients. Like several other studies [37,32,33] we could not use a correction factor for readmissions and transfers to other hospitals, since such a correction factor is available for Germany, but not for Austria. The Austrian association of insurance companies made preliminary data accessible, which allowed estimation of hip fracture, related readmission rate in (personal communication, data available on request). According to these data there were 30,418 hospital admissions due to hip fracture in 24,856 patients in the years 2006 and 2007, yielding a mean rate of 19% of patient transfers and readmissions. Analyses of German data indicate a transfer and readmission rate of 30% in 2001 and 11% in 2006, respectively [38,39]. However, a systematic evaluation stratifying for age, sex, and region covering a longer time period is required. Thus, we cannot exclude that transfers and readmissions affect our results. Furthermore, the analysis of hip fracture trends over the 10-year study period is unlikely to be influenced by lack of a correction factor. Another limitation might be the difference between Austria and Germany in change from ICD 9 to ICD 10. In Austria, this change took place one year later. However, the classification of hip fractures is clearly categorised in both versions and misclassifications are rather unlikely.

## Conclusion

This is the first study comparing hip fracture incidence rates between Austria and Germany using pooled original data from both countries and the same methods. The results indicate a 30% higher overall incidence rate of hip fractures in Austria. Despite the observed difference in incidence levels, the hip fracture incidence trend was comparable. In both countries, the secular trends between 1995 and 2004 indicate neither a levelling-off nor a decrease but a similar increase of hip fracture incidence rates.

High quality prospective studies are warranted to confirm our results.

## Competing interests

The authors declare that they have no competing interests.

## Authors' contributions

EM, GM and AI initiated the study. EM and AI developed the study protocol. EM and AI coordinated the data analysis, and interpreted the results with help of the other authors. BH performed the statistical analysis. EM wrote the paper. All authors commented on paper drafts. AI is the guarantor for the paper.

## Pre-publication history

The pre-publication history for this paper can be accessed here:

http://www.biomedcentral.com/1471-2458/10/46/prepub
